# Distressed Democrats and relaxed Republicans? Partisanship and mental health during the COVID-19 pandemic

**DOI:** 10.1371/journal.pone.0266562

**Published:** 2022-04-21

**Authors:** Sean Bock, Landon Schnabel

**Affiliations:** 1 Department of Sociology, Harvard University, Cambridge, MA, United States of America; 2 Department of Sociology, Cornell University, Ithaca, NY, United States of America; Texas State University, UNITED STATES

## Abstract

The COVID-19 pandemic was a potent stressor, yielding unprecedented levels of mental distress. However, public health responses and personal reactions to the pandemic were politically polarized, with Democrats highlighting and Republicans downplaying its severity. Did Republicans subsequently experience as much mental distress as Democrats during the COVID-19 pandemic? This study examines partisan patterns in mental health outcomes at three time points throughout the pandemic. Results demonstrate a clear partisan distress gap, with Democrats consistently reporting worse mental health than Republicans. Trend data suggest that the 2020 pandemic patterns are a continuation and exacerbation of an existing partisan distress gap. Consideration of race, however, demonstrates a widening partisan distress gap, specific to white Americans. Among white Americans, therefore, Democrats experienced a substantially greater increase in distress in response to the pandemic than Republicans.

## Introduction

The COVID-19 pandemic prompted a mental health crisis, effecting unprecedented levels of mental distress in the United States and around the world [1–6]. For instance, the percentage of Americans who say they are “not too happy” has reached the highest levels since the General Social Survey first started asking about happiness in the early 1970s [7]. Unprecedented distress is unsurprising given the hardships faced during the pandemic, where fear of the virus itself joined with existential insecurity, disrupted routines, and general societal upheaval to create a unique array of stressors [8, 9]. Compounding matters was the fact that the public health measures necessary to prevent the spread of the virus robbed individuals of the social connection central to their wellbeing [10–13].

Responses to the pandemic, the sacrifices to normalcy needed to curb it, and even whether it was a real threat to be concerned about were quickly politicized by politicians, pundits, and the public alike [14–18]. Emerging research on partisanship and the pandemic in the United States demonstrates vastly different responses and approaches to whether the pandemic is real, something to stress about, and important enough to make a person limit their in-person connections, disrupt their routine, and constrain their daily life up to and including isolation [15, 17, 19–21]. It is possible, therefore, that although a widespread pandemic presumably exposes everyone to generally similar stressors, partisanship may cause people to experience and respond to those stressors quite differently.

Under normal circumstances, mental health is socially structured by a number of factors, such as race, income, age, and politics [22, 23]. Stressors are unequally distributed across society, with, for example, racial minorities experiencing disproportionate levels of exposure to distressing conditions [24–26]. We would not expect distress patterns during the pandemic to be homogeneous, and while everyone is experiencing the pandemic they are not experiencing or responding to it in the same way. Even in the absence of a highly politicized pandemic, political views are a consistent predictor of happiness and subjective wellbeing. Research consistently demonstrates that political liberals experience less subjective wellbeing—typically measured with general happiness or life satisfaction—than political conservatives [27–29]. Explanations as to the specific mechanisms for this pattern vary, but most studies point to ideological and personality differences such as a sense of certainty and control, rather than other factors, such as demographic makeup [22, 28, 30–32]. Regardless of the specific mechanisms, the data consistently demonstrate a political gap in mental health, whereby political liberals on average are more predisposed to have worse subjective wellbeing than political conservatives. And it appears that politicized stressors, such as mass shootings, can widen partisan gaps in wellbeing by having greater negative impacts on the emotional well-being of Democrats [33]. In light of politicized responses to the pandemic—where Democrats are more likely to see the pandemic as a threat, more likely to socially distance, and presumably more likely to face a perfect storm of threats to mental health (e.g., existential insecurity, social disconnectedness, and disrupted routine)—did this predisposition lead to higher levels of distress among Democrats in 2020?

Emerging research on responses to the pandemic provide every reason to suspect that distress should be patterned by partisanship. For example, Democrats demonstrated greater support for mask-wearing, lockdown measures, and vaccinations compared to Republicans [14–18, 34–36]. Simply, Democrats, on average, have displayed more concern for the virus, and this difference in concern may have translated into disparities in negative mental health consequences during the pandemic. The result may have been a partisan physical vs. mental health trade-off, with Democrats experiencing more disruption of routine and social isolation to reduce the risk of physical illness and Republicans risking physical health to maintain normalcy and sustain social connections.

Given the known political disparities in subjective wellbeing, along with the differential levels of concern regarding the pandemic, we predict Democrats experienced worse mental health (i.e., greater levels of distress) over the course of 2020.

This study tests this idea by employing three waves of the 2020 NORC COVID-19 Response Tracking Study (Wave 1 was collected in May, Wave 2 in late June through early July, and Wave 3 in late July through early August). We analyze responses to a range of mental health questions to determine whether Democrats experienced more distress than Republicans during the pandemic. We further examine several items from the 2018 NORC General Social Survey (GSS) wave that match outcomes in the NORC COVID-19 Response Tracking Study, allowing us to asses pre- and post-levels of distress between partisans. Finally, we decompose the patterns by race because race is highly correlated with partisanship, typical experiences with stressors, and mental health. It is possible that, for example, Black Americans—who are by far the most consistently Democrat—were already experiencing exceptionally high levels of stressors and were thus better prepared to cope with stressors because of more experience with them or simply already at a ceiling of distress with less room to move than whites.

## Materials and methods

### Data

The main data are from the 2020 NORC COVID Response Tracking Survey, fielded at the University of Chicago. The sample is 2,000 respondents, selected using AmeriSpeak-NORC‘s probability-based panel, and was collected online and over the phone in English and Spanish. Respondents were interviewed in three waves, between May and August of 2020 (Wave 1: 5/21–5/29; Wave 2: 6/22–7/6; Wave 3: 7/22–8/10. The survey asks a range of questions about Americans’ views on and experiences with the COVID-19 pandemic.

The survey was designed to continue several mental health items that were fielded in the General Social Survey (GSS) before 2020. We take advantage of this feature and combine GSS data with the COVID Response Tracking Survey data to analyze trends on select items, allowing us to observe distress levels before and during the pandemic. To our knowledge, this combined data set offers the most complete picture of trends in mental distress, the impact of the COVID-19 pandemic on these trends, and the mediating effect of political partisanship. See S1 Table in S1 Appendix for weighted descriptive statistics.

### Ethics statement

We did not seek IRB approval for this study, as all data (both the GSS and the NORC COVID Response Survey) used are publicly available and de-identified. Therefore, this project does not involve research on human subjects, as defined by Harvard University’s Institutional Review Board (see https://cuhs.harvard.edu/do-you-need-irb-review-and-why). For more information on the collection of data in both surveys, visit https://www.norc.org.

#### Dependent variables

We measure experienced distress with several variables, including: emotional problems, fatigue, mental health, general unhappiness, quality of life, a COVID-19 reaction scale, a feeling scale, a loneliness scale, and a stress scale. See S2 Table in S1 Appendix for question wording and measurement.

#### Independent variables

Our key independent variable is a three-category measure of partisan identification: Democrat, Independent, and Republican. Each partisan category includes those leaning toward one party or the other. We also include a number of standard socio-demographic variables and COVID-19 impact variables in our multivariable regression models. See S3 Table in S1 Appendix for question wording and measurement.

#### Analytic strategy

**Fig 1:**
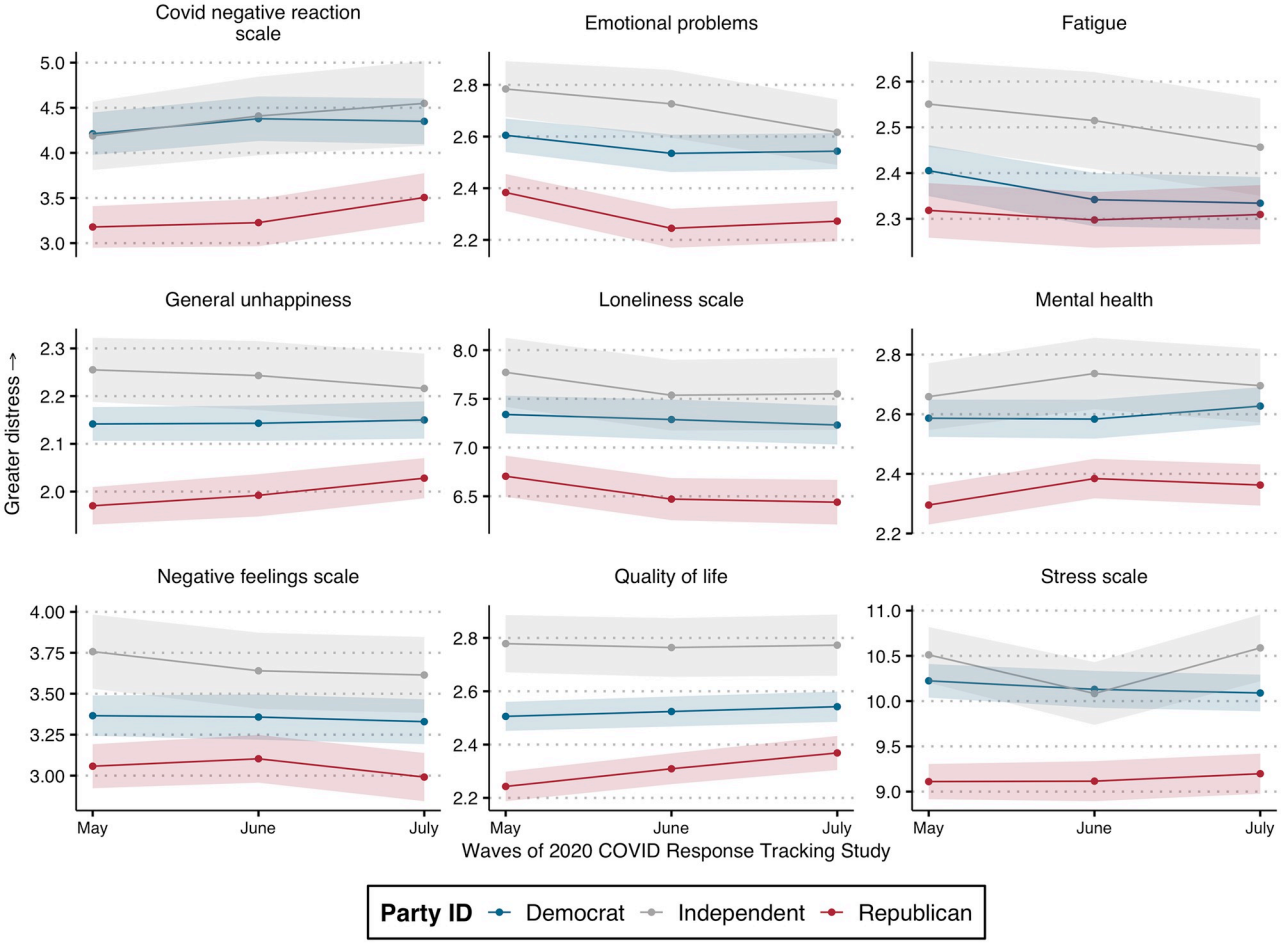
Partisan distress patterns over 2020. Data are from NORC COVID-19 response survey. Note: Points indicate mean response by group with 95% confidence bands. Higher values = greater distress.

**Fig 2:**
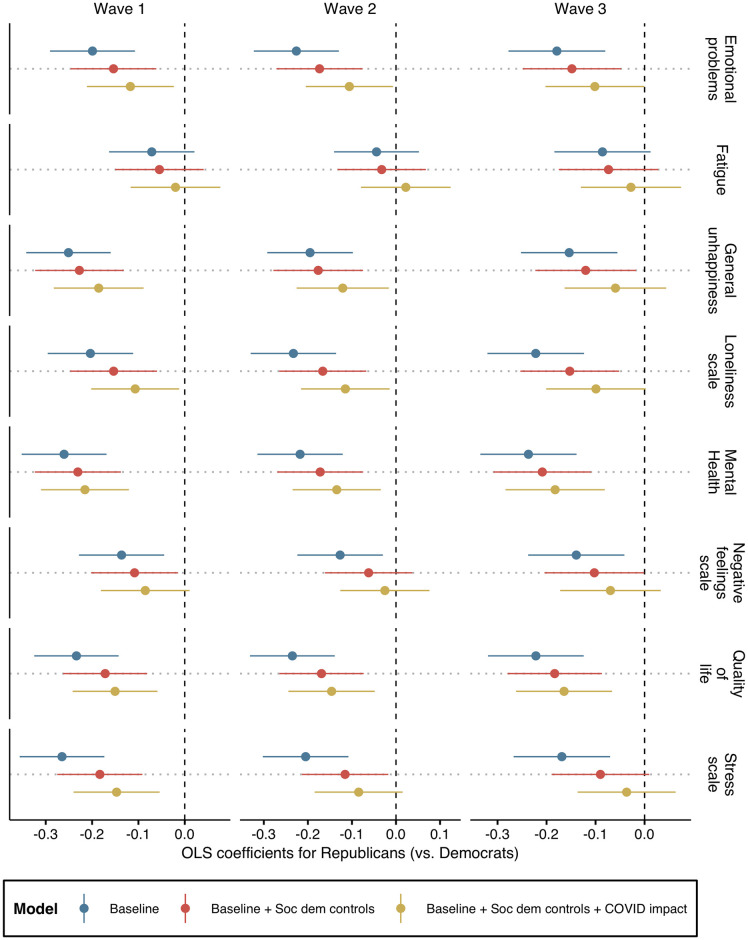
OLS coefficient plot with 95% confidence intervals. Note: Data from NORC COVID Response Tracking Study. Positive estimates indicate more distress for Republicans compared to Democrats. Outcomes are standardized in order to compare association sizes between estimates.

**Fig 3:**
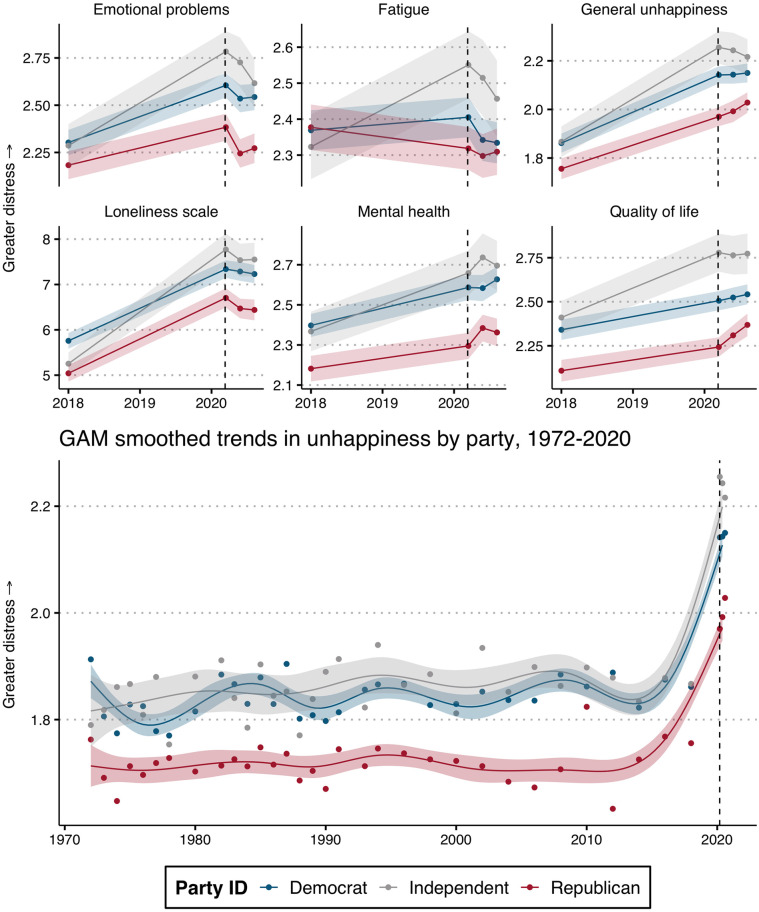
Over-time partisan distress trends. Data are from GSS and 2020 NORC COVID-19 response survey. Note: Points indicate mean response by group in each year. Higher values = greater distress. Dashed line indicates beginning of pandemic. Confidence bands demonstrate uncertainty at the 95% level. Smoothed trends (bottom panel) are predicted from a Generalized Additive Model (GAM) with restricted maximum likelihood estimation.

**Fig 4:**
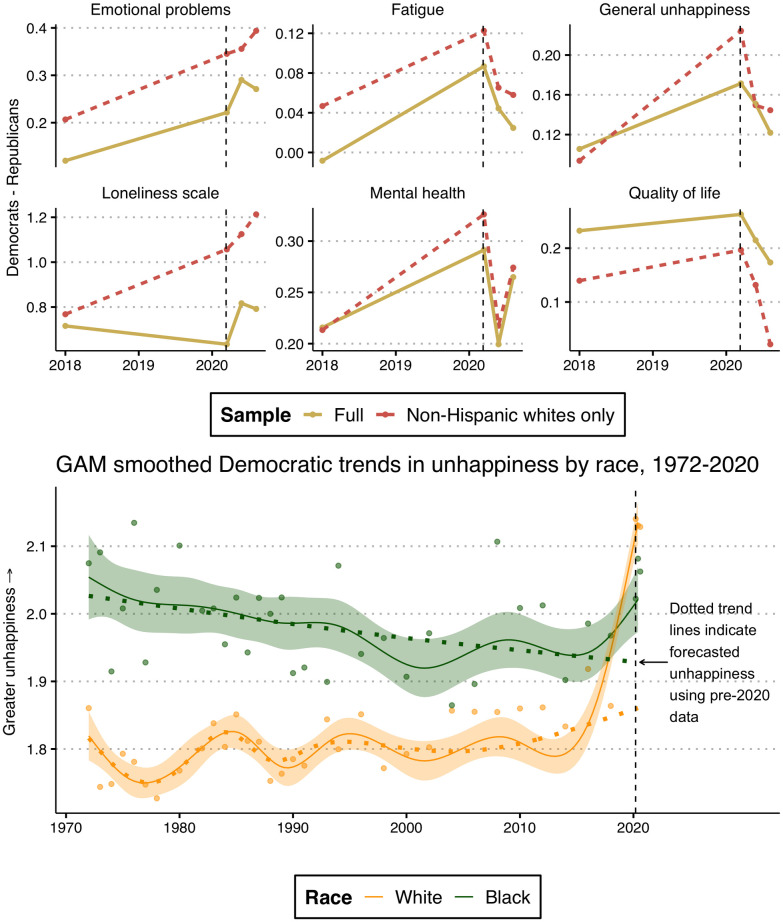
Decomposed over-time trends. Top panel displays trends in the partisan gap with a full sample and a sample restricted to whites only. Bottom panel displays smoothed over-time trends in unhappiness among white and black Democrats. Note: Because the GSS did not ask respondents about ethnicity until 2000, ‘white’ include both Hispanic and non-Hispanic whites before 2000. Higher values = greater distress. Dashed line indicates beginning of pandemic. Confidence bands demonstrate uncertainty at the 95% level. Smoothed trends (bottom panel) are predicted from a Generalized Additive Model (GAM) with restricted maximum likelihood estimation.

The main baseline patterns (Figs 1, 3 and 4) are all calculated using weighted means for each outcome by partisan ID. We also run several OLS regression models predicting distress. The first model type is a baseline regression, only including partisan ID. The second model adds controls for typical socio-demographic variables. And, the third model adds COVID-19 impact variables. Each model type is run for all distress outcomes. To allow for potential over-time differences during 2020, we run models separately across each wave. Fig 2 displays coefficients for all 72 models. Full OLS results are included in S4–S6 Tables in S1 Appendix.

See S1 Appendix for more information on the data and variables. All analyses were performed in R (version 4.0.4).

## Results

### Partisan distress gap

Fig 1 plots mean distress scores for partisans across three waves of the 2020 data. Across all measures and waves, there is a clear partisan gap in distress, with Republicans consistently reporting lower distress levels compared to Democrats. Moreover, Independents—a disparate group comprising 16% of the sample once leaners are categorized—consistently reported high distress levels compared to both Democrats and Republicans. Of note, the over-time patterns appear to vary by partisans: While Democrats held fairly consistent distress levels across most measures, Republicans reported increased distress over the course of 2020–though never reaching the distress levels of Democrats. This may reflect increasing concern over the pandemic among Republicans following the early months.

Fig 1 confirms the expected partisan distress patterns: Democrats consistently displayed higher levels of distress in the pandemic, and this distress gap persisted throughout all three waves. What explains these patterns? Although the baseline gap between Democrats and Republicans is clear, this pattern could be driven by factors other than partisanship or ideology. For instance, demographic differences between party members may explain the partisan distress gap. A separate but related potential confounder could be personal experience with the pandemic: we know the pandemic affected Americans differently across geographic location and among different social groups. Geographic disparities were especially apparent early in the pandemic, and thus these effects could be playing an important role for the patterns found in Wave 1 of the data, for example. To control for the potential impact of confounders, the next set of results displays estimates from multivariable models, predicting the partisan distress gap while holding other variables constant.

### Multivariable models

Fig 2 plots coefficients with accompanying 95% confidence intervals across all outcomes for each wave. The coefficients estimate the difference in distress levels for Republicans compared to Democrats. Positive estimates would indicate that Republicans experienced more distress on a given measure. The first model (in blue) are baseline, bivariate estimates. The second model (red) introduces socio-demographic controls. And, the third model (yellow) adds controls for personal COVID impact including living in a “hotspot” with high infection rates, exposure to the virus, having a family member diagnosed, family impact from the virus, negative economic impact due to the virus, and level of information exposure regarding the virus (see Suporting Information for more information on COVID impact variables).

The baseline model formally estimates the partisan gap presented in Fig 1 with 95% confidence intervals. Across all measures in each wave, the coefficients for Republicans are negative and all but fatigue are significant. Are these partisan patterns explained by standard socio-demographic factors? Overall, estimates from the second model suggest not: the direction of all coefficients remain the same and the sizes of the associations are attenuated but similar and the general pattern remains. The coefficients are further attenuated in the third model once controls for personal impact by the pandemic are included. Apart from the estimate for fatigue in Wave 2, all coefficients remain negative. However, more estimates are now non-significant across the three waves compared to the other models. Moreover, there appears to be an over-time dynamic, with only two of the eight coefficients remaining significant by Wave 3.

On the one hand, it is not surprising that the increased number of parameters in the third model has led to fewer coefficients remaining significant. On the other hand, the over-time patterns could suggest the partisan gap was increasingly explained by a sorting of experiences with COVID, whereby Democrats were disproportionately impacted personally by the pandemic (e.g., the first “hotspots” were cosmopolitan areas with high population density and global interconnectedness, which are predominantly occupied by Democrats). Cross-model comparisons are not significantly different, though, so we are cautious to make strong claims about these patterns.

Taken together, results from the multivariable models confirm the expected partisan distress gap during the pandemic, and this gap cannot be completely explained by the makeup of the parties, nor partisans’ differential exposure to the pandemic across the three waves. Although the observed partisan gap during the pandemic is substantial and consistent, we should compare it to patterns before the pandemic to determine the extent to which it is the function of a partisan response to the pandemic or reflective of an already existing gap. To ascertain the effect of the pandemic on partisan distress patterns, we matched several outcomes with those present in the 2018 GSS. With these matched data prior to the pandemic, we can assess the extent to which the gap between Democrats and Republicans emerged or grew from 2018 to 2020.

### Trends in distress

Fig 3 is a duplication of Fig 1 with the addition of the 2018 GSS data. First, there was a clear increase in distress levels among all partisans from 2018 to 2020, with the exception of fatigue, which has a more nuanced pattern. Across all measures (again, except for fatigue), a clear partisan gap already existed in 2018, indicating that the pandemic did not create the partisan distress gap out of thin air. Supplementary analyses of additional data confirm both the overall increase in distress among respondents and the partisan gap during the pandemic (see S3 and S4 Figs in S1 Appendix). Although it is clear that the pandemic did not create the gap, there is some limited evidence that it may have exacerbated existing differences between Republicans and Democrats (as well as Independents): the partisan gap between Republicans and Democrats widened between 2018 and 2020 on emotional problems, happiness, and mental health, though these changes are not significant.

Although trends are limited to 2018 and 2020 for most measures, the GSS has been fielding the “General Happiness” item since 1974. The bottom panel of Fig 3 displays smoothed partisan trends on happiness going back several decades. These trends highlight the long-running, consistent partisan gap and the effect of the pandemic, which starkly increased unhappiness overall. For instance, the mean unhappiness level for Republicans in 2020 is higher than levels reported from Democrats and Independents from any previous wave.

These over-time results indicate a stable and consistent partisan distress gap. Rather than an increased distinction between Democrats and Republicans, distress levels during the pandemic appear to reflect a pre-existing partisan gap, with a raised baseline level of distress for all Americans. Given the extremely partisan responses to the pandemic, this is a surprising result.

### Race and trends in partisan gap

Why was there no significant increase in the partisan distress gap during the pandemic? Put differently, why did Democrats not further distinguish themselves from Republicans in terms of their distress levels? To better understand partisan responses to the pandemic–especially among Democrats–it is important to consider racial patterns. The top panel of Fig 4 plots distress gap trends for the full sample and a reduced sample of white Americans. (See S1 Fig in S1 Appendix for within-party distress trends by race.) These trends help explain the previous results. Whereas the full sample trends suggested that the pandemic did not yield partisan distress, Fig 4 shows that the partisan gap grew among white Americans on several outcomes, including loneliness, emotional problems, happiness, and mental health (though the mental health gap collapsed during later months of 2020). (Note: “Whites” include non-Hispanic whites in the top panel of Fig 4. In the bottom panel, however, “Whites” includes both Hispanic and non-Hispanic whites before 2000, because the GSS did not ask respondents about ethnicity until the 2000 wave. Only non-Hispanic whites are included in 2000 and beyond. Patterns remain similar when including both non-Hispanic and Hispanic whites in the full time series).

To further contextualize these patterns, we evaluated over-time trends in unhappiness among white and black Democrats (bottom panel of Fig 4). To emphasize the impact of the pandemic, the dotted trend lines indicate forecasted levels of unhappiness in 2020 with pre-2020 data. The results reveal a clear racial pattern among Democrats and help to explain the general patterns found in the top panel of Fig 4. While there was a sharp increase in unhappiness from 2018 to 2020 among white Democrats—reaching the highest levels of unhappiness by far over the past 50 years—black Democrats experienced a much subtler bump in distress, reaching levels similar to previous GSS waves and lower than several others. These trends reveal that while the partisan distress gap was once heavily influenced by the disproportionate levels of distress among black Democrats, white Democrats were the clear drivers of the gap in 2020. Notably, while black Democrats did not experience as sharp of an increase in distress, the pandemic appears to have reversed a 50-year decline in mental distress for this group. Prior to the pandemic, the happiness gap between white and black Democrats had been slowly shrinking due primarily to increasing happiness among black Democrats but also a slow rise in unhappiness among white Democrats. The pandemic rapidly closed and even reversed the gap, such that white Democrats were unhappier than black Democrats for the first time since the beginning of the General Social Survey. As a result, the distress level among Democrats during the pandemic was mitigated by the relative resiliency among black Democrats, blunting what would have otherwise been a clear increased partisan distress gap as a result of the pandemic. Supplementary analyses demonstrate that in a counterfactual scenario in which black democrats experienced the same increase in distress from 2018 to 2020 as white democrats, the partisan gap in happiness grew substantially. (See S2 Fig in S1 Appendix).

### Limitations

There are several limitations to the analyses that should be noted. First, this study relies on repeated cross-sectional data, which has important implications for our findings. Because we cannot observe the same participants over time, we do not know if partisan patterns in distress are caused by actual changes *among* partisans, or if the makeup of Democrats and Republicans has changed over time, driving the observed patterns [37]. Ideally, demographic controls in multivariable models can account for this possibility to some extent, but unobserved variables likely remain that may bias our estimates. We test for this possibility by using GSS Panel data (2016–2020), allowing us to track wellbeing before and after the pandemic among the same respondents (See S3 and S4 Figs in S1 Appendix). These data allow us to accurately asses changes among partisans, as well as control for other time-invariant variables. While the GSS 2016–2020 Panel only includes the happiness and loneliness items, for at least these items, the same general patterns found in the main analyses hold, suggesting that changes in the composition of Democrats and Republicans are not driving the partisan distress patterns we see in 2020. Second, apart from the COVID-19-specific measures, we cannot preclude the possibility that partisan patterns in wellbeing during 2020 were impacted by other salient events, such as the volatile 2020 presidential election and racial injustice movements. Third, interpreting the associations between partisanship and wellbeing as causal require further assumptions that are not met in our research design. For instance, because we cannot assign partisan ID (i.e., the “treatment”) to respondents, we are not able to say whether partisan affiliation “causes” wellbeing, or if baseline wellbeing causes individuals to sort into one party versus the other. However, the goal of this paper is not necessarily to establish a causal relationship between partisan ID and wellbeing. Rather, we seek to describe patterns of wellbeing in the population, noting how partisans diverged during the COVID-19 pandemic and how those patterns relate to historical trends.

## Conclusion

The COVID-19 pandemic acted as a powerful stressor, leading to increased distress among Americans. Distress during the pandemic was politically polarized, however, such that Democrats reported consistently higher distress levels compared to Republicans, suggesting a clear partisan distress gap. This gap did not simply emerge as a result of the pandemic, but rather reflects a pre-existing partisan distress gap. Yet, marked intra-party variation in experiences with the pandemic results in a more nuanced story: White Democrats experienced the largest increase in distress along several measures from the pandemic, which produced a widening distress gap among white partisans. Further, over-time data show the pandemic accelerated a long-running trend of a closing of the racial happiness gap among Democrats. Whereas the partisan gap used to be disproportionately driven by the unhappiness of black Democrats, it is now driven primarily by the unhappiness of white Democrats. These patterns highlight the importance of race for trends in partisanship and polarization more generally.

The results point to several possible extensions. The data for this study are from the summer of 2020, before widespread vaccine roll out. As the pandemic wears on, we are experiencing a mental health crisis. Isolation, distress about the present, and uncertainty about the future—not to mention lost friends and family—are taking a toll. Partisan vaccine uptake further highlights the partisan nature of pandemic response, but it may have changed the equation in ways that should be examined in future research. With vaccines, Democrats may be able to regain some of the normalcy and connections they lost in isolation. And serious illness and death have become visibly partisan with the vaccines, and the greater loss experienced among Republicans, and perhaps eventual acceptance of COVID-19 as a real threat and the pandemic something to worry about, may fuel greater distress among them than in the past. Beyond the partisan nature of mental wellbeing in the pandemic, we hope this study will motivate future research on race (and ethnicity) in these processes more generally. These data were effective for highlighting race trends between black and white Americans over decades and future research should further disentangle racial and ethnic trends in partisanship and distress among and between more groups.

## Supporting information

S1 Appendix (PDF)
