# Supplementary material for: Distressed Democrats and relaxed Republicans? Partisanship and mental health during the COVID-19 pandemic
Source: PLoS One. 2022 Apr 21;17(4):e0266562. doi: 10.1371/journal.pone.0266562 (PMC9022799; doi:10.1371/journal.pone.0266562)
Supplement: S1 Appendix — (PDF) [file pone.0266562.s001.pdf]

Distressed Democrats and Relaxed Republicans? Partisanship and Mental Health During the COVID-19 Pandemic: SI-Appendix *

Sean Bock *Harvard University*
Landon Schnabel *Cornell University*

This is the appendix to the article entitled 'Distressed Democrats and Relaxed Republicans? Partisanship and Mental Health During the COVID-19 Pandemic'. We provide additional information on our data, variables, and modeling, as well as supplemental analyses.

Table S1: Weighted descriptive statistics across three waves of the NORC COVID-19 Response Survey

	Wave 1: (5/21/20 - 5/29/20)			Wave 2: (6/22/20 - 7/6/20)			Wave 3: (7/22/20 - 8/10/20)		
Variable	N	Mean	SD	N	Mean	SD	N	Mean	SD
Party I.D.	2271			2006			2002		
... Democrat	1064	47%		942	48%		950	49%	
... Independent	351	16%		301	16%		310	16%	
... Republican	856	37%		763	36%		742	35%	
General unhappiness	2263			2007			1996		
... Not too happy	325	14%		287	14%		286	14%	
... Pretty happy	1422	63%		1239	62%		1222	61%	
... Very happy	516	23%		481	24%		488	25%	
Emotional problems	2269			2008			2001		
... Never	469	19%		492	24%		464	22%	
... Not very often	676	30%		592	29%		624	31%	
... Pretty often	1048	47%		845	42%		842	42%	
... Very often	76	4%		79	5%		71	4%	
Fatigue	2273			2004			2005		
... Never	378	16%		345	16%		350	17%	
... Not very often	911	39%		837	43%		837	43%	
... Pretty often	952	43%		788	39%		779	39%	
... Very often	32	2%		34	2%		39	2%	
Mental health	2274			2007			2001		
... Never	385	17%		301	14%		305	14%	
... Not very often	863	37%		786	38%		779	37%	
... Pretty often	966	43%		865	44%		861	45%	
... Very often	60	3%		55	3%		56	4%	
Quality of life	2276			2010			2006		
... Never	268	13%		220	11%		202	11%	

Current version*: April 13, 2022; **Corresponding author: lschnabel@cornell.edu.

Table S1: Weighted descriptive statistics across three waves of the NORC COVID-19 Response Survey *(continued)*

Variable	N	Mean	SD	N	Mean	SD	N	Mean	SD
... Not very often	1021	43%		878	42%		905	42%	
... Pretty often	947	42%		878	45%		862	44%	
... Very often	40	2%		34	2%		37	2%	
COVID-19 negative reaction scale	2204	3.82	3.72	1957	3.96	3.84	1962	4.08	3.95
Negative feelings scale	2220	3.31	2.06	1973	3.31	2.12	1954	3.25	2.11
Lonliness scale	2260	7.17	3.24	1996	7.03	3.18	1982	7	3.2
Stress scale	2249	9.86	3.04	1985	9.76	3.18	1973	9.85	3.2
Education	2279			2012			2007		
... HS or less	528	38%		443	38%		427	38%	
... Some college	935	28%		814	28%		820	28%	
... BA or more	816	34%		755	34%		760	34%	
Income	2279			2012			2007		
... Less than $50,000	1040	49%		886	50%		879	49%	
... $50,000 to under $100,00	785	32%		717	32%		710	32%	
... $100,000 or more	454	19%		409	18%		418	19%	
Employment status	2279			2012			2007		
... Not employed	896	42%		775	42%		750	42%	
... Employed	1383	58%		1237	58%		1257	58%	
Martial status	2279			2012			2007		
... Not married	904	41%		775	41%		766	41%	
... Married/co-habitating	1375	59%		1237	59%		1241	59%	
Race	2279			2012			2007		
... White	1500	63%		1348	63%		1334	63%	
... Black	237	12%		195	12%		194	12%	
... Hispanic	370	17%		317	17%		330	17%	
... Other	172	9%		152	9%		149	9%	
Region	2279			2012			2007		
... Northeast	359	17%		313	17%		304	17%	
... Midwest	577	21%		516	21%		522	21%	
... South	804	38%		700	38%		691	38%	
... West	539	24%		483	24%		490	24%	
Age	2279			2012			2007		
... 18-29	263	21%		224	21%		231	21%	
... 30-39	525	17%		466	17%		481	17%	
... 40-59	722	32%		650	32%		663	32%	
... 60-64	248	8%		225	8%		222	8%	
... 65 or older	521	21%		447	21%		410	21%	
COVID-19: info	2272			2010			2005		
... Never	30	1%		38	2%		29	1%	
... Rarely	261	11%		304	16%		262	13%	

Table S1: Weighted descriptive statistics across three waves of the NORC COVID-19 Response Survey *(continued)*

Variable	N	Mean	SD	N	Mean	SD	N	Mean	SD
... Occasionally	837	37%		803	40%		714	35%	
... Often	888	39%		706	34%		805	41%	
... Most of the time	256	11%		159	8%		195	10%	
COVID-19: hotspot	2279			2012			2007		
... Not a hotspot	1772	82%		1572	81%		1554	80%	
... Hotspot	507	18%		440	19%		453	20%	
COVID-19: exposure	2279			2012			2007		
... No	1980	87%		1748	86%		1689	85%	
... Yes	299	13%		264	14%		318	15%	
COVID-19: family diag.	2279			2012			2007		
... No	2084	91%		1856	92%		1786	88%	
... Yes	195	9%		156	8%		221	12%	
COVID-19: economic impact	2264			1995			1994		
... No	1651	72%		1489	74%		1487	75%	
... Yes	613	28%		506	26%		507	25%	
COVID-19: family impact	2226	1.09	1.34	1986	0.91	1.31	1975	0.89	1.27

Variables

Table S2: Description of distress variables

Variable	Question	Measurement
Emotional problems	‘In the past seven days, how often have you been bothered by emotional problems such as feeling anxious, depressed or irritable?’	1 = ‘Never’, 5 = ‘Always’
Fatigue	’In the past seven days, how would you rate your fatigue on average?’	1 = ‘None’, 5 = ‘Very severe’
Mental health	’In general, how would you rate your mental health, including your mood and your ability to think?’	1 = ‘Poor’, 5 = ‘Excellent’

Table S2: Description of distress variables *(continued)*

Variable	Question	Measurement
General unhappiness	’Taken all together, how would you say things are these days—would you say that you are very happy, pretty happy, or not too happy?’	1 = ’Very happy’, ’2 = ’Not too happy’
Quality of life	’In general, would you say your quality of life is...	1 = ’Excellent’, 5 = ’Poor’
Covid reaction scale	’Please indicate whether or not each of the following have happened to you since the outbreak of the coronavirus/COVID-19 pandemic... ’ [Didn’t feel like eating] [Smoked more than usual] [Had headaches] [Had an upset stomach] [Cried] [Had trouble getting to sleep] [Felt very nervous and tense] [Felt like getting drunk] [Felt more tired than usual] [Felt dizzy at times] [Lost my temper more than usual] [Hands sweat and felt damp and clammy] [Had rapid heart beats] [Felt sort of dazed and numb] [Kept forgetting things]	0 = ’No’, 1 = ’Yes’ (Scale created from sum of responses)

Table S2: Description of distress variables *(continued)*

Variable	Question	Measurement
Feel scale	'During the past few weeks did you ever feel...' [Particularly excited or interested in something] [So restless that you couldn't sit long in a chair] [Proud because someonoe complicated you on something you had done] [Very lonely or remote from other people] [Pleased about having accomplished something] [Bored] [On top of the world] [Depressed or very unhappy] [That things were going your way] [Upset because someone criticized you]	0 = 'No', 1 = 'Yes' (Scale created from sum of responses)
Loneliness scale	'How often in the past 4 weeks have you felt that you lack companionship?', 'How often in the past 4 weeks have you felt that you are isolated from others?', 'How often in the past 4 weeks have you felt that you are left out?'	1 = 'Never', 5 = Very often' (Scale created from sum of responses)

Table S2: Description of distress variables *(continued)*

Variable	Question	Measurement
Stress scale	'In the past 4 weeks, how often have you felt that you were unable to contorl the importnat things in your life?', 'In the past 4 weeks, how often have you felt confident about your ability to handle your personal problems?', 'In the past 4 weeks, how often have you felt that things were going your way?'	1 = 'Never', 5 = Very often' (Scale created from sum of responses)

Table S3: Description of independent variables

Variable	Measurement
Party Identification	1 = 'Democrat', 2 = 'Independnet', 3 = 'Republican'
Education	1 = High school or less, 2 = Some college, 3 = BA or more
Income	1 = Less than $50,000, 2 = $50,000 to under $100,000, 3 = $100,000 or more
Employment status	0 = 'Not employed', 1 = 'Employed'
Marital status	0 = 'Not married', 1 = 'Married/co-habitating'
Race/ethnicity	1 = White, 3 = Black, 3 = Hispanic, 4 = Other
Geographic region	1 = Northeast, 2 = Midwest, 3 = South, 4 = West
Age	18-29, 30-39, 40-59, 60-64, 65 or older
COVID-19 info: 'How much are you watching, reading, or talking about the coronavirus/COVID-19?'	1 = 'Never', 5 = 'Most of the time'

Table S3: Description of independent variables *(continued)*

Variable	Measurement
COVID-19 hotspot: R lived in area with high COVID-19 infection and death rates at time of interview	0 = 'Not a hotspot', 1 = 'Hotspot'
COVID-19 exposure: 'During the past month, have you been exposed to someone likely to have the coronavirus/COVID-19?'	'0 = 'No', 1 = 'Yes'
COVID-19 family diagnosis: 'During hte past month, has anyone in your family been diagnosed with the coronavirus/COVID-19?'	0 = 'No', 1 = 'Yes'
COVID-19 family impact scale: 'During the past month, have any of the following happened to your family members because of the coronavirus/COVID-19?' [Lost or been laid off from job] [Reduced ability to earn money] [Passed away]	0 = 'No', 1 = 'Yes' (Scale created from sum of responses)
COVID-19 economic impact: 'In what ways, if any, has the coronavirus affected your job, income, or finances: You lost income due to a workplace closure or reduced hours'	0 = 'No', 1 = 'Yes'

Table S4: Multivariable OLS regressions, predicting distress: Wave 1

	Emotional problems	Fatigue	Happiness	Mental health	Lonliness	Quality of life	Feel	Stress	Covid-19 reaction
(Intercept)	3.241***	2.816***	2.212***	3.055***	9.189***	2.480***	4.429***	10.939***	4.409***
	(0.207)	(0.179)	(0.125)	(0.196)	(0.624)	(0.169)	(0.416)	(0.585)	(0.728)
Independent	0.083	0.043	0.079*	0.041	0.208	0.155**	0.242+	0.183	−0.213
	(0.063)	(0.055)	(0.037)	(0.060)	(0.192)	(0.052)	(0.125)	(0.178)	(0.211)
Republican	−0.130**	−0.031	−0.116***	−0.215***	−0.342*	−0.145***	−0.175+	−0.446**	−0.784***
	(0.050)	(0.043)	(0.029)	(0.047)	(0.151)	(0.041)	(0.098)	(0.140)	(0.166)
Some college	0.065	0.023	0.051	0.054	0.396*	−0.032	0.223*	−0.021	0.379*
	(0.057)	(0.049)	(0.034)	(0.054)	(0.173)	(0.047)	(0.113)	(0.161)	(0.191)
BA or more	−0.031	−0.083	−0.021	−0.158**	0.025	−0.298***	−0.008	−0.526**	0.112
	(0.065)	(0.056)	(0.038)	(0.061)	(0.195)	(0.053)	(0.128)	(0.181)	(0.214)
$100K or more	−0.266***	−0.209***	−0.127**	−0.306***	−0.409*	−0.371***	−0.360**	−0.661***	−0.678**
	(0.067)	(0.057)	(0.039)	(0.063)	(0.201)	(0.054)	(0.131)	(0.186)	(0.221)
Employed	−0.280***	−0.228***	−0.108***	−0.249***	−0.862***	−0.212***	−0.527***	−0.620***	−0.695***
	(0.051)	(0.044)	(0.030)	(0.048)	(0.154)	(0.042)	(0.101)	(0.143)	(0.170)
Black	−0.354***	−0.288***	−0.124**	−0.320***	−1.274***	0.012	−0.785***	−0.568**	−1.481***
	(0.078)	(0.067)	(0.045)	(0.073)	(0.234)	(0.063)	(0.152)	(0.216)	(0.257)
Hispanic	−0.221***	−0.156**	0.027	−0.117+	−0.426*	0.087	−0.529***	−0.094	−0.462*
	(0.067)	(0.057)	(0.039)	(0.063)	(0.201)	(0.054)	(0.131)	(0.187)	(0.221)
Other	−0.161+	−0.021	0.070	−0.012	0.087	0.078	−0.072	0.289	−0.178
	(0.084)	(0.072)	(0.049)	(0.079)	(0.251)	(0.068)	(0.165)	(0.234)	(0.274)
Midwest	−0.005	−0.013	0.006	0.056	−0.051	0.035	0.148	0.098	0.149
	(0.071)	(0.061)	(0.042)	(0.067)	(0.215)	(0.058)	(0.140)	(0.199)	(0.235)
South	−0.035	0.077	−0.045	−0.013	−0.237	0.058	0.107	0.166	−0.100
	(0.069)	(0.060)	(0.040)	(0.065)	(0.209)	(0.056)	(0.136)	(0.194)	(0.228)
West	−0.054	−0.026	−0.053	−0.061	−0.285	0.008	−0.031	−0.280	−0.157
	(0.072)	(0.062)	(0.042)	(0.068)	(0.217)	(0.059)	(0.141)	(0.200)	(0.236)
30-39	−0.040	−0.016	0.066	−0.078	0.061	0.129*	0.168	−0.041	0.089
	(0.078)	(0.067)	(0.046)	(0.074)	(0.236)	(0.064)	(0.153)	(0.218)	(0.258)
40-59	−0.330***	−0.158*	0.109*	−0.102	−0.386+	0.271***	−0.167	−0.713***	−1.146***

Table S4: Multivariable OLS regressions, predicting distress:
Wave 1 *(continued)*

	Emotional problems	Fatigue	Happiness	Mental health	Lonliness	Quality of life	Feel	Stress	Covid-19 reaction
	(0.075)	(0.065)	(0.044)	(0.071)	(0.227)	(0.062)	(0.148)	(0.210)	(0.249)
60-64	−0.577***	−0.345***	0.056	−0.430***	−0.861**	0.191*	−0.655***	−1.795***	−2.318***
	(0.092)	(0.079)	(0.054)	(0.087)	(0.277)	(0.075)	(0.181)	(0.257)	(0.304)
65 or older	−0.909***	−0.399***	−0.001	−0.590***	−1.419***	0.038	−1.185***	−2.322***	−3.115***
	(0.084)	(0.072)	(0.049)	(0.079)	(0.252)	(0.068)	(0.165)	(0.234)	(0.277)
Rarely	−0.166	−0.185	−0.082	0.027	−0.563	0.140	−0.313	0.149	0.300
	(0.193)	(0.166)	(0.117)	(0.182)	(0.580)	(0.158)	(0.386)	(0.545)	(0.684)
Often	−0.001	−0.064	−0.035	0.039	−0.192	0.103	−0.323	0.244	1.238+
	(0.187)	(0.161)	(0.113)	(0.177)	(0.562)	(0.153)	(0.374)	(0.528)	(0.664)
Occasionally	−0.068	−0.103	−0.078	0.018	−0.398	0.104	−0.396	0.166	0.599
	(0.186)	(0.160)	(0.113)	(0.176)	(0.561)	(0.152)	(0.374)	(0.527)	(0.663)
Most of the time	0.132	0.017	0.062	0.050	0.396	0.223	−0.095	0.493	1.833**
	(0.194)	(0.167)	(0.117)	(0.184)	(0.584)	(0.159)	(0.389)	(0.549)	(0.688)
Hotspot	0.008	0.005	0.058+	0.004	0.213	0.086+	0.081	0.194	−0.054
	(0.057)	(0.049)	(0.033)	(0.054)	(0.172)	(0.046)	(0.112)	(0.159)	(0.189)
Family diagnosis	−0.124	0.024	−0.079	−0.157+	−0.061	−0.096	0.258	−0.421	−0.435
	(0.093)	(0.080)	(0.054)	(0.088)	(0.284)	(0.076)	(0.181)	(0.260)	(0.308)
Impacted economically	0.143**	0.088+	0.067*	−0.018	0.314*	0.157***	0.243*	0.284+	0.806***
	(0.052)	(0.045)	(0.031)	(0.050)	(0.158)	(0.043)	(0.103)	(0.146)	(0.172)
Family impacted	0.094***	0.064***	0.055***	0.088***	0.269***	0.083***	0.113**	0.322***	0.575***
	(0.019)	(0.016)	(0.011)	(0.018)	(0.058)	(0.016)	(0.037)	(0.053)	(0.063)
Num.Obs.	2192	2195	2189	2195	2185	2197	2151	2175	2138
R2	0.150	0.084	0.087	0.127	0.135	0.181	0.112	0.162	0.243
R2 Adj.	0.139	0.072	0.075	0.117	0.124	0.170	0.101	0.151	0.233
AIC	6204.6	5559.5	3855.9	5971.5	11 000.7	5333.2	8951.9	10 605.3	11 113.2
BIC	6369.7	5724.6	4020.9	6136.6	11 165.7	5498.4	9116.4	10 770.2	11 277.5
Log.Lik.	−3073.309	−2750.742	−1898.929	−2956.755	−5471.352	−2637.612	−4446.938	−5273.669	−5527.579

Table S4: Multivariable OLS regressions, predicting distress: Wave 1 *(continued)*

	Emotional problems	Fatigue	Happiness	Mental health	Lonliness	Quality of life	Feel	Stress	Covid-19 reaction
F	14.096	7.350	7.597	11.723	12.426	17.701	9.962	15.362	25.082

+ $p < 0.1$, * $p < 0.05$, ** $p < 0.01$, *** $p < 0.001$

Table S5: Multivariable OLS regressions, predicting distress: Wave 2

	Emotional problems	Fatigue	Happiness	Mental health	Lonliness	Quality of life	Feel	Stress	Covid-19 reaction
(Intercept)	3.086***	2.603***	2.112***	3.285***	9.272***	2.863***	5.153***	12.270***	5.170***
	(0.200)	(0.170)	(0.117)	(0.182)	(0.587)	(0.158)	(0.393)	(0.574)	(0.672)
Independent	0.173*	0.132*	0.070+	0.048	0.332	0.119*	0.216	−0.051	−0.154
	(0.069)	(0.059)	(0.041)	(0.063)	(0.204)	(0.055)	(0.137)	(0.201)	(0.234)
Republican	−0.120*	−0.007	−0.089**	−0.146**	−0.378*	−0.139**	−0.052	−0.327*	−0.569**
	(0.054)	(0.046)	(0.032)	(0.049)	(0.160)	(0.043)	(0.108)	(0.157)	(0.183)
Some college	0.174**	0.092+	0.053	−0.056	0.484**	−0.042	0.223+	−0.034	0.485*
	(0.063)	(0.054)	(0.037)	(0.057)	(0.185)	(0.050)	(0.125)	(0.182)	(0.213)
BA or more	0.085	−0.061	−0.021	−0.290***	0.031	−0.283***	−0.085	−0.410*	0.236
	(0.070)	(0.059)	(0.041)	(0.064)	(0.206)	(0.055)	(0.139)	(0.202)	(0.235)
$100K or more	−0.294***	−0.273***	−0.182***	−0.360***	−0.340	−0.451***	−0.395**	−0.833***	−0.651**
	(0.072)	(0.061)	(0.042)	(0.065)	(0.211)	(0.056)	(0.142)	(0.207)	(0.240)
Employed	−0.281***	−0.207***	−0.073*	−0.199***	−0.801***	−0.188***	−0.695***	−0.777***	−0.754***
	(0.055)	(0.047)	(0.032)	(0.050)	(0.162)	(0.043)	(0.109)	(0.159)	(0.185)
Black	−0.468***	−0.243***	−0.114*	−0.296***	−1.172***	0.002	−0.507**	−0.750**	−1.642***
	(0.086)	(0.073)	(0.050)	(0.078)	(0.254)	(0.068)	(0.170)	(0.248)	(0.288)
Hispanic	−0.146*	−0.184**	−0.023	−0.113+	−0.269	0.067	−0.272+	−0.128	−0.796**
	(0.073)	(0.062)	(0.043)	(0.066)	(0.214)	(0.057)	(0.145)	(0.210)	(0.245)
Other	−0.104	−0.026	0.011	−0.074	−0.038	0.088	−0.069	0.054	−0.465
	(0.090)	(0.076)	(0.053)	(0.082)	(0.264)	(0.071)	(0.178)	(0.259)	(0.299)
Midwest	−0.028	−0.008	0.051	0.159*	−0.011	0.104+	0.113	0.070	0.137
	(0.077)	(0.065)	(0.045)	(0.070)	(0.227)	(0.061)	(0.152)	(0.222)	(0.258)
South	−0.025	0.017	0.018	0.050	0.061	0.062	0.019	0.044	0.283
	(0.075)	(0.064)	(0.044)	(0.068)	(0.221)	(0.059)	(0.148)	(0.216)	(0.251)
West	−0.023	0.007	−0.009	0.100	−0.174	0.062	0.028	−0.076	0.213
	(0.078)	(0.066)	(0.045)	(0.070)	(0.229)	(0.061)	(0.153)	(0.223)	(0.260)
30-39	−0.103	−0.020	0.067	−0.119	−0.022	0.162*	0.024	−0.092	−0.419
	(0.085)	(0.072)	(0.050)	(0.078)	(0.251)	(0.067)	(0.169)	(0.246)	(0.286)
40-59	−0.341***	−0.014	0.152**	−0.119	−0.249	0.299***	−0.301+	−0.563*	−1.581***

Table S5: Multivariable OLS regressions, predicting distress: Wave 2 *(continued)*

	Emotional problems	Fatigue	Happiness	Mental health	Lonliness	Quality of life	Feel	Stress	Covid-19 reaction
	(0.083)	(0.070)	(0.049)	(0.075)	(0.243)	(0.065)	(0.164)	(0.238)	(0.277)
60-64	−0.651***	−0.200*	0.137*	−0.443***	−0.832**	0.194*	−0.820***	−1.733***	−3.023***
	(0.100)	(0.085)	(0.059)	(0.091)	(0.296)	(0.079)	(0.199)	(0.290)	(0.338)
65 or older	−0.878***	−0.343***	0.005	−0.652***	−1.565***	0.026	−1.293***	−2.279***	−3.399***
	(0.092)	(0.079)	(0.054)	(0.084)	(0.272)	(0.073)	(0.184)	(0.267)	(0.311)
Rarely	−0.273	−0.195	−0.095	−0.243	−1.225*	−0.279*	−1.157***	−1.323**	−0.046
	(0.177)	(0.150)	(0.104)	(0.161)	(0.520)	(0.140)	(0.348)	(0.509)	(0.597)
Often	0.000	0.015	0.042	−0.111	−0.470	−0.214	−0.882**	−0.965+	0.973+
	(0.173)	(0.147)	(0.102)	(0.158)	(0.509)	(0.137)	(0.341)	(0.498)	(0.584)
Occasionally	−0.161	−0.055	−0.048	−0.177	−1.033*	−0.240+	−0.995**	−1.255*	0.392
	(0.172)	(0.146)	(0.101)	(0.156)	(0.504)	(0.135)	(0.337)	(0.493)	(0.577)
Most of the time	0.180	0.087	0.092	0.008	−0.052	−0.199	−0.397	−0.875	2.030**
	(0.188)	(0.160)	(0.111)	(0.172)	(0.554)	(0.149)	(0.370)	(0.542)	(0.634)
Hotspot	0.030	0.065	0.094**	0.086	0.223	0.049	0.106	0.466**	0.178
	(0.062)	(0.052)	(0.036)	(0.056)	(0.182)	(0.049)	(0.122)	(0.179)	(0.208)
Family diagnosis	−0.161	0.003	−0.090	−0.033	0.084	−0.170*	−0.027	−0.377	−0.578
	(0.102)	(0.087)	(0.060)	(0.093)	(0.302)	(0.081)	(0.204)	(0.295)	(0.353)
Impacted economically	0.136*	0.065	0.071*	0.051	0.503**	0.135**	0.312**	0.430*	0.443*
	(0.058)	(0.050)	(0.034)	(0.053)	(0.171)	(0.046)	(0.115)	(0.168)	(0.195)
Family impacted	0.140***	0.105***	0.037**	0.057**	0.237***	0.063***	0.146***	0.356***	0.728***
	(0.022)	(0.019)	(0.013)	(0.020)	(0.064)	(0.017)	(0.043)	(0.063)	(0.074)
Num.Obs.	1960	1956	1960	1959	1950	1962	1930	1940	1913
R2	0.169	0.100	0.077	0.146	0.139	0.169	0.122	0.152	0.234
R2 Adj.	0.157	0.087	0.064	0.134	0.127	0.157	0.109	0.140	0.223
AIC	5631.9	4979.1	3548.4	5257.1	9804.1	4705.7	8151.9	9667.3	10 090.6
BIC	5793.8	5140.9	3710.2	5418.9	9965.8	4867.6	8313.3	9828.8	10 251.8
Log.Lik.	−2786.955	−2460.539	−1745.186	−2599.528	−4873.071	−2323.852	−4046.930	−4804.636	−5016.312

Table S5: Multivariable OLS regressions, predicting distress: Wave 2 *(continued)*

	Emotional problems	Fatigue	Happiness	Mental health	Lonliness	Quality of life	Feel	Stress	Covid-19 reaction
F	14.541	7.898	5.981	12.183	11.490	14.532	9.784	12.662	21.357

+ p < 0.1, * p < 0.05, ** p < 0.01, *** p < 0.001

Table S6: Multivariable OLS regressions, predicting distress: Wave 3

	Emotional problems	Fatigue	Happiness	Mental health	Lonliness	Quality of life	Feel	Stress	Covid-19 reaction
(Intercept)	2.636***	2.632***	1.833***	2.782***	7.677***	2.332***	3.473***	10.534***	5.337***
	(0.225)	(0.190)	(0.132)	(0.204)	(0.675)	(0.177)	(0.463)	(0.640)	(0.762)
Independent	0.003	0.007	0.050	−0.025	0.266	0.094+	0.167	0.367+	−0.409+
	(0.069)	(0.058)	(0.040)	(0.063)	(0.205)	(0.054)	(0.138)	(0.196)	(0.235)
Republican	−0.097+	−0.013	−0.038	−0.181***	−0.284+	−0.137**	−0.144	−0.139	−0.614***
	(0.054)	(0.046)	(0.032)	(0.049)	(0.162)	(0.043)	(0.108)	(0.155)	(0.185)
Some college	0.068	0.042	0.002	−0.050	0.224	−0.021	0.026	−0.331+	0.452*
	(0.064)	(0.054)	(0.038)	(0.058)	(0.191)	(0.050)	(0.129)	(0.184)	(0.220)
BA or more	−0.009	−0.112+	−0.073+	−0.258***	0.020	−0.257***	−0.218	−0.540**	0.177
	(0.071)	(0.060)	(0.042)	(0.064)	(0.211)	(0.056)	(0.142)	(0.203)	(0.242)
$100K or more	−0.250***	−0.257***	−0.142***	−0.339***	−0.504*	−0.490***	−0.352*	−0.907***	−0.765**
	(0.072)	(0.061)	(0.042)	(0.066)	(0.214)	(0.057)	(0.143)	(0.206)	(0.244)
Employed	−0.220***	−0.223***	−0.076*	−0.192***	−0.657***	−0.192***	−0.366***	−0.608***	−0.756***
	(0.056)	(0.047)	(0.033)	(0.051)	(0.166)	(0.044)	(0.111)	(0.159)	(0.190)
Black	−0.482***	−0.296***	−0.082	−0.284***	−1.116***	−0.075	−0.704***	−0.656**	−1.849***
	(0.087)	(0.073)	(0.051)	(0.078)	(0.258)	(0.068)	(0.174)	(0.247)	(0.297)
Hispanic	−0.166*	−0.127*	0.059	−0.138*	−0.220	0.033	−0.192	−0.258	−0.824***
	(0.073)	(0.061)	(0.043)	(0.066)	(0.216)	(0.057)	(0.144)	(0.207)	(0.249)
Other	−0.189*	−0.054	0.083	−0.027	−0.142	0.148*	−0.289	0.028	−0.854**
	(0.092)	(0.077)	(0.053)	(0.083)	(0.271)	(0.072)	(0.181)	(0.260)	(0.310)
Midwest	−0.092	−0.057	0.083+	0.004	−0.082	0.068	0.084	−0.096	−0.014
	(0.078)	(0.066)	(0.046)	(0.071)	(0.231)	(0.061)	(0.155)	(0.223)	(0.265)
South	−0.084	−0.074	−0.016	−0.101	−0.061	0.040	0.069	0.118	−0.141
	(0.076)	(0.064)	(0.044)	(0.069)	(0.226)	(0.060)	(0.151)	(0.218)	(0.259)
West	−0.080	−0.078	0.020	−0.041	−0.190	0.024	0.186	−0.112	0.114
	(0.078)	(0.065)	(0.045)	(0.071)	(0.231)	(0.061)	(0.154)	(0.223)	(0.266)
30-39	−0.031	0.013	0.118*	−0.050	0.339	0.163*	0.111	−0.119	−0.028
	(0.085)	(0.071)	(0.050)	(0.077)	(0.252)	(0.067)	(0.168)	(0.242)	(0.289)
40-59	−0.267**	−0.083	0.176***	−0.084	−0.213	0.359***	−0.116	−0.592*	−1.264***

Table S6: Multivariable OLS regressions, predicting distress: Wave 3 *(continued)*

	Emotional problems	Fatigue	Happiness	Mental health	Lonliness	Quality of life	Feel	Stress	Covid-19 reaction
	(0.082)	(0.069)	(0.048)	(0.074)	(0.242)	(0.064)	(0.162)	(0.233)	(0.279)
60-64	−0.553***	−0.212*	0.126*	−0.310***	−0.474	0.268***	−0.539**	−1.704***	−2.527***
	(0.101)	(0.085)	(0.059)	(0.091)	(0.298)	(0.079)	(0.199)	(0.287)	(0.341)
65 or older	−0.798***	−0.383***	0.037	−0.573***	−1.119***	0.062	−1.002***	−2.339***	−3.221***
	(0.093)	(0.078)	(0.054)	(0.084)	(0.275)	(0.073)	(0.184)	(0.265)	(0.316)
Rarely	0.284	−0.010	0.174	0.354+	0.331	0.279+	0.324	0.467	−0.375
	(0.204)	(0.172)	(0.119)	(0.185)	(0.614)	(0.160)	(0.422)	(0.579)	(0.690)
Often	0.510*	0.118	0.348**	0.442*	1.014+	0.336*	0.456	1.074+	0.748
	(0.198)	(0.167)	(0.116)	(0.179)	(0.596)	(0.155)	(0.410)	(0.562)	(0.668)
Occasionally	0.467*	0.109	0.224+	0.393*	0.590	0.353*	0.404	0.873	0.286
	(0.198)	(0.167)	(0.116)	(0.179)	(0.595)	(0.155)	(0.410)	(0.562)	(0.668)
Most of the time	0.621**	0.142	0.415***	0.463*	1.205+	0.400*	0.721+	1.416*	1.589*
	(0.208)	(0.175)	(0.122)	(0.189)	(0.626)	(0.164)	(0.429)	(0.592)	(0.704)
Hotspot	0.037	0.066	0.043	0.036	0.264	0.118*	0.213+	0.314+	0.173
	(0.062)	(0.052)	(0.036)	(0.056)	(0.183)	(0.048)	(0.122)	(0.175)	(0.210)
Family diagnosis	−0.085	0.046	−0.004	0.000	0.200	−0.109	0.085	−0.126	−0.249
	(0.091)	(0.076)	(0.053)	(0.082)	(0.270)	(0.071)	(0.182)	(0.260)	(0.311)
Impacted economically	0.106+	0.043	0.047	0.032	0.293+	0.085+	0.293**	0.361*	0.759***
	(0.057)	(0.048)	(0.033)	(0.052)	(0.168)	(0.045)	(0.113)	(0.163)	(0.194)
Family impacted	0.139***	0.106***	0.043**	0.067***	0.226***	0.053**	0.185***	0.351***	0.717***
	(0.022)	(0.019)	(0.013)	(0.020)	(0.067)	(0.018)	(0.045)	(0.064)	(0.076)
Num.Obs.	1957	1960	1956	1956	1941	1961	1914	1938	1921
R2	0.137	0.092	0.079	0.122	0.119	0.170	0.092	0.149	0.231
R2 Adj.	0.125	0.079	0.066	0.110	0.107	0.159	0.079	0.137	0.220
AIC	5654.2	4989.0	3548.5	5266.6	9817.2	4717.0	8116.7	9642.1	10 224.0
BIC	5816.0	5150.9	3710.2	5428.3	9978.7	4878.8	8277.8	9803.6	10 385.3
Log.Lik.	−2798.105	−2465.514	−1745.233	−2604.279	−4879.577	−2329.494	−4029.330	−4792.059	−5083.025

Table S6: Multivariable OLS regressions, predicting distress: Wave 3 *(continued)*

	Emotional problems	Fatigue	Happiness	Mental health	Lonliness	Quality of life	Feel	Stress	Covid-19 reaction
F	11.337	7.208	6.090	9.924	9.611	14.707	7.098	12.372	21.057
+ p < 0.1, * p < 0.05, ** p < 0.01, *** p < 0.001									

Supplementary figures

Counterfactual unhappiness gap trends

Figure 2 displays the partisan gap in unhappiness over time, including a counterfactual scenario. The red line indicates what the gap would have been in 2020 if Black Democrats experienced the same level of distress as White Democrats. The counterfactual highlights the impact of the relatively low increase in distress among Black Democrats in 2020 on the partisan unhappiness gap.

GSS 2016-2020 Panel

The GSS released the 2020 companion data to the 2016 and 2018 panel waves in May of 2021. Respondents were matched from either the 2016 and 2018 waves in 2020, but no respondents were interviewed in all three waves. 2020 interviews were conducted in September of 2020, meaning that these data were collected at least one month after the last wave of the NORC COVID-19 Response Survey data. Figures 3 and 4 plot partisan trends for two samples. Figure 3 shows overall trends of unhappiness with combined samples. Figure 4 plots unhappiness trends, limited to respondents that maintained the same partisan I.D. between their respective first and second interviews. Figure 4 allows us to control for the potential of party I.D. switching affecting partisan trends in unhappiness. The results provide additional evidence of increased distress during the pandemic for all respondents. Further, these results provide some evidence that the partisan gap may have increased at later points in 2020 (compared to trends seen in the NORC COVID-19 Response Survey data). Given small sample sizes—especially once broken down into sub-party groups for those who held consistent party I.D.s—we are hesitant to draw any strong conclusions about within-party trends.

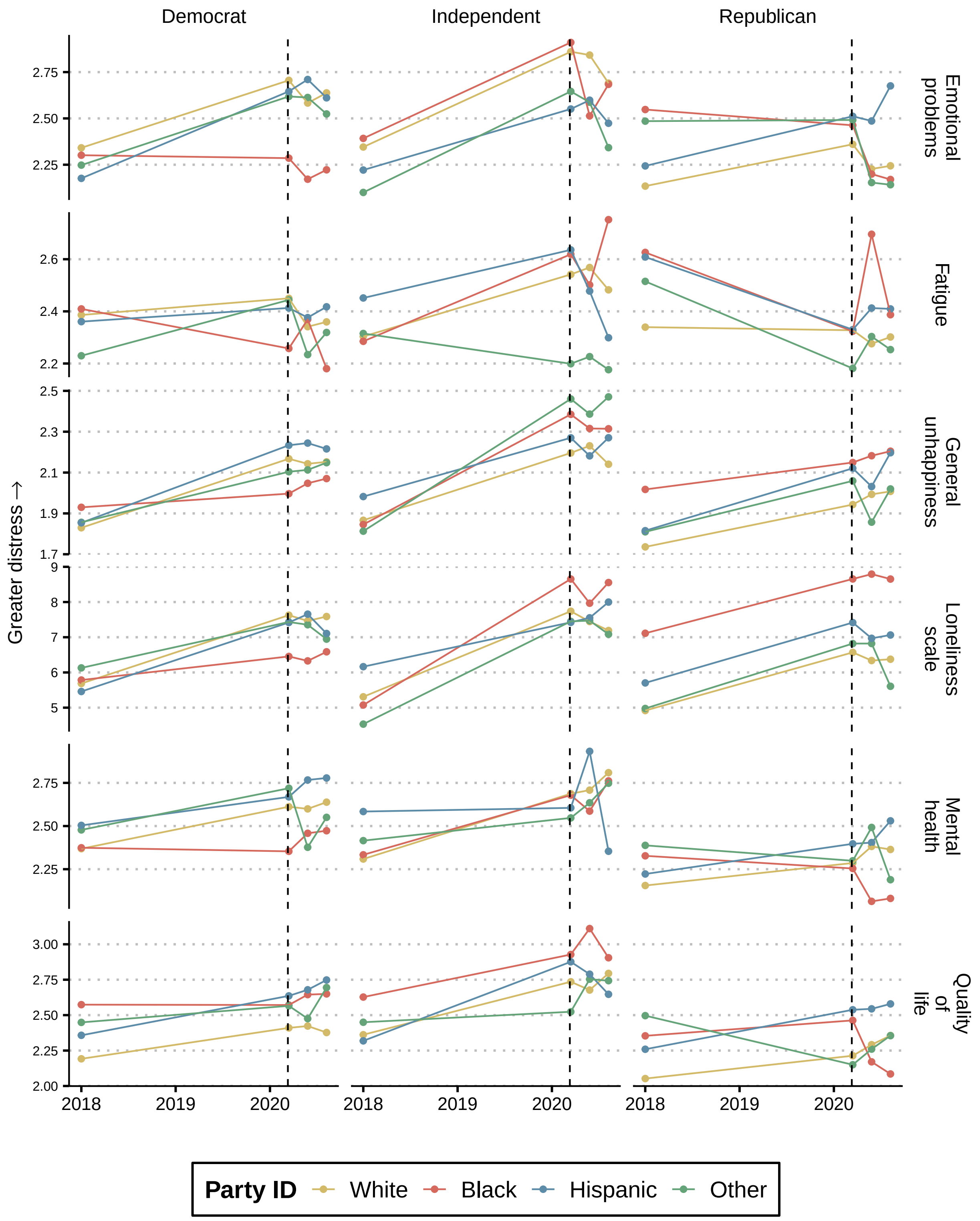

Note: Points indicate mean response by group. Higher values = greater distress. Dashed line indicates beginning of pandemic.'White' includes non−hispanic whites only.

Figure S1: Distress trends: PartyXRace

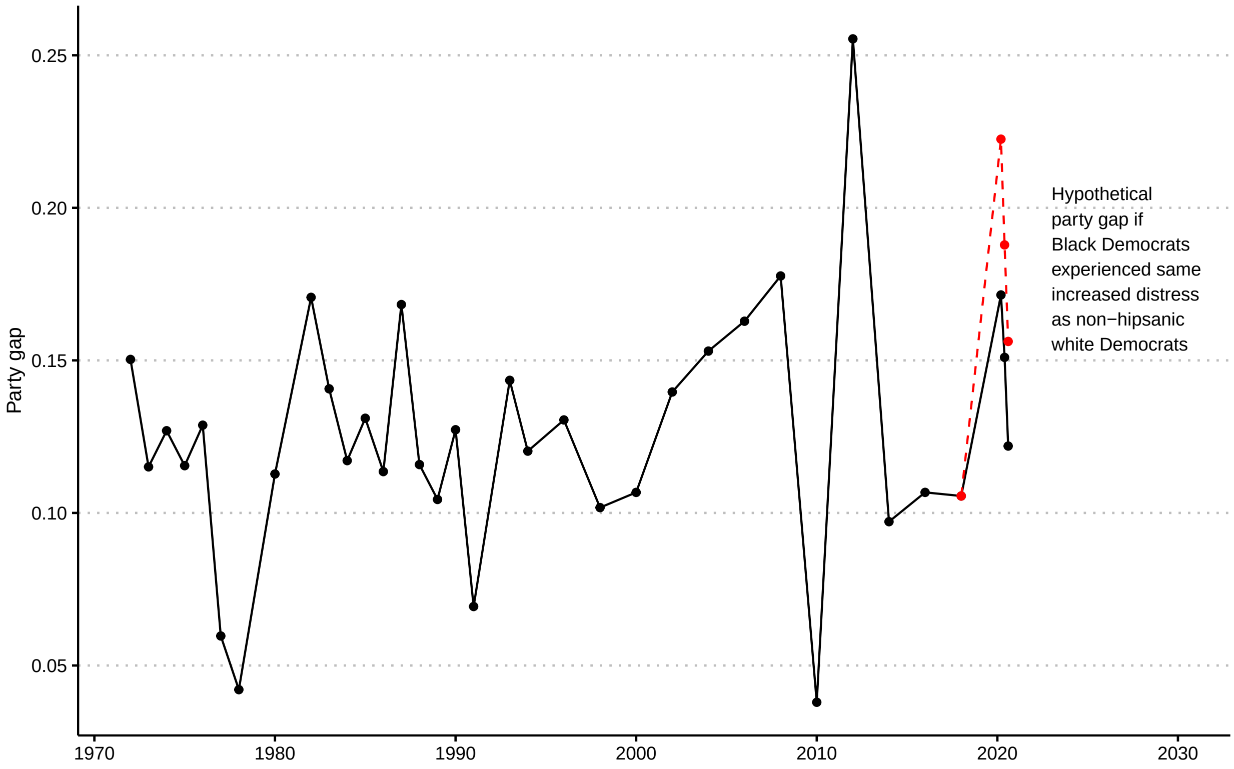

Figure S2: Counterfactual party gap if black Democrats experienced same distress as white Democrats

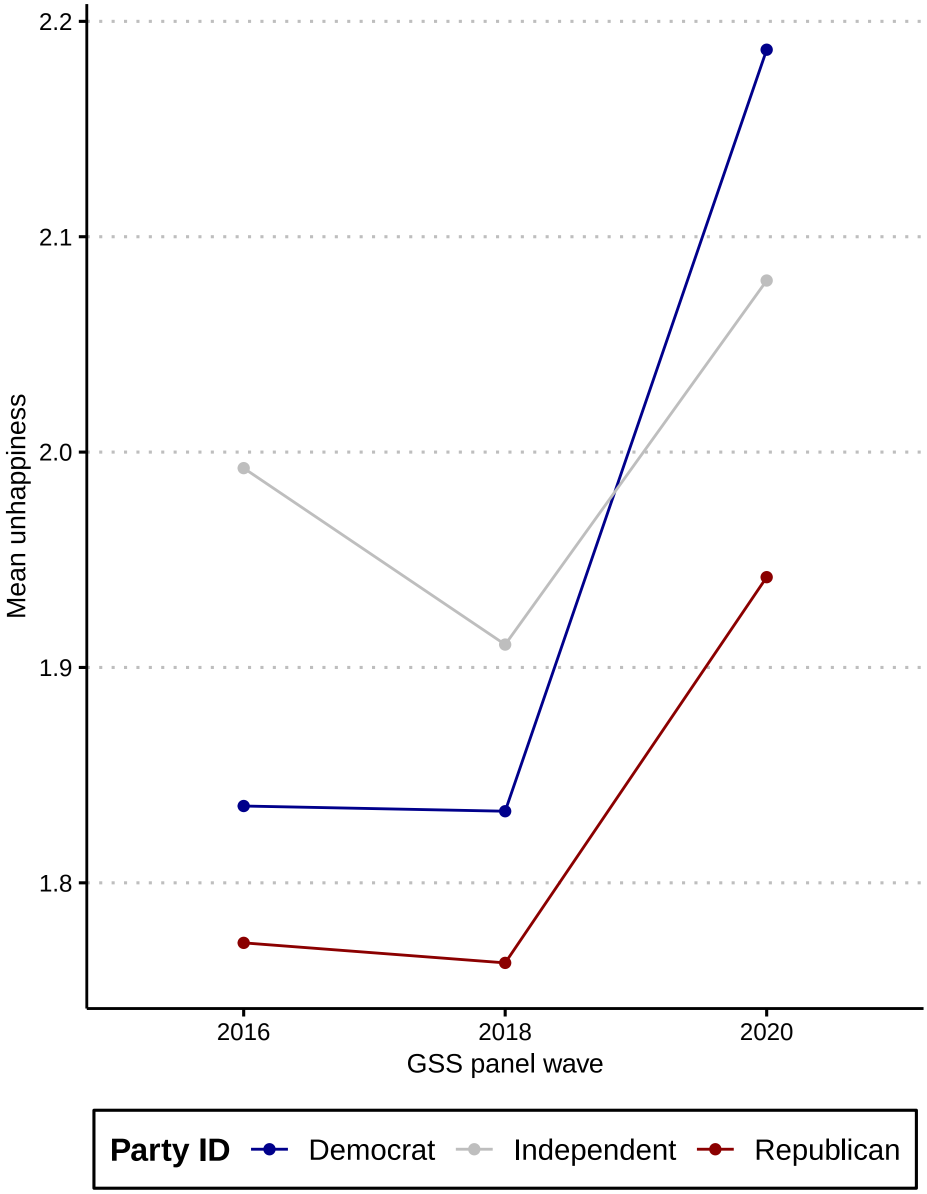

Figure S3: Unhappiness partisan trends with GSS panel data, 2016-2020. Trends include combined samples.

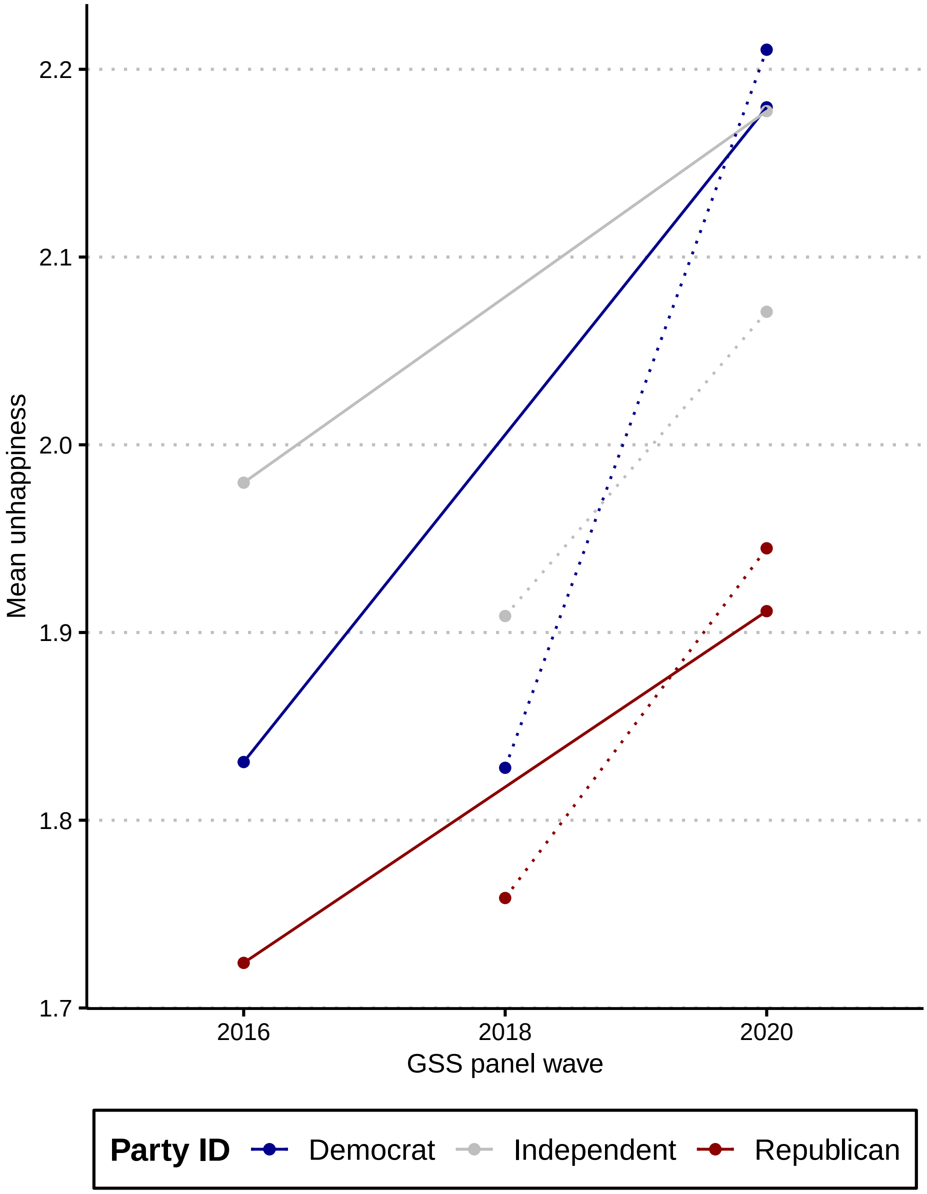

Figure S4: Unhappiness partisan trends with matched GSS panel data. Sample limited to respondents with consistent partisan I.D. across both waves.
